# Residual nitrite and nitrate in processed meats and meat analogues in the United States

**DOI:** 10.1038/s41598-025-87563-x

**Published:** 2025-01-25

**Authors:** Siyuan Sheng, Erin M. Silva, Rodrigo Tarté, James R. Claus

**Affiliations:** 1 Department of Animal & Dairy Sciences, University of Wisconsin-Madison, Madison, WI 53706 USA; 2 Department of Plant Pathology, University of Wisconsin-Madison, Madison, WI 53706 USA; 3 Department of Animal Science, Iowa State University, Ames, IA 50011 USA

**Keywords:** Nitrite, Nitrate, Cured meats color, Processed meats, Meat analogue, Risk factors, Analytical chemistry, Bioanalytical chemistry

## Abstract

Residual nitrite (NO_2_^−^) and nitrate (NO_3_^−^) have been widely studied in the past few decades for their function to improve processed meat quality and their impact on human health^1–4^. In this study we examined how the residual nitrite and nitrate (NO_x_^−^) content of major classes of processed meats products (*n* = 1132) produced locally from three regions (East Coast, Midwest and West Coast) and plant protein-based meat analogues (*n* = 53) available at retail in the United States was influenced by their composition, processing, and geographical attributes. We also conducted time-dependent depletion studies and observed different patterns of NO_x_^−^ depletion and conversion during processing and storage and correlated them with product quality. Together, our results reveal a comprehensive prospective of NO_x_^−^ content in processed meats and meat analogues. The NO_2_^−^ in processed meats and meat analogues averaged (± standard error; minimum and maximum value in parentheses) 13.7 ± 0.62 (0.0-214.5) and 1.7 ± 0.34 (0.0–11.0), respectively, and the NO_3_^−^ in processed meats and meat analogues averaged 32.6 ± 0.90 (2.0–205.9) and 7.2 ± 0.56 (4.0-25.3) ppm, respectively.

**Supplementary Information:**

The online version contains supplementary material available at 10.1038/s41598-025-87563-x.

## Introduction

The curing ingredients nitrite (NO_2_^-^) and nitrate (NO_3_^-^) are commonly used as antimicrobials to inhibit *Clostridium botulinum*, limit growth of spoilage organisms, retard lipid peroxidation, and provide the unique flavor and color of cured meats^5–7^. In the United States, regulations limit the amount of added sodium nitrite and nitrate to ingoing concentrations of 200 and 700 ppm for whole muscle, and 156 and 1718 ppm for comminuted products, respectively^8^. NO_3_^-^ in processed meats could be inadvertently introduced from various sources including water, non-meat ingredients, processing aids, and raw meat. Moreover, NO_2_^-^ and NO_3_^-^ could interconvert with each other as well as interact with other nitrogen-based compounds and food components during storage and processing^9,10^.

According to the International Agency for Research on Cancer (IARC), an agency of the World Health Organization (WHO), they have classified processed meats as a Group 1 carcinogen^11^. Relative to this risk, the current acceptable daily intake for NO_2_^-^ is 0–0.07 milligrams per kilogram of body weight (BW), and 0–3.7 mg/kg BW for NO_3_^-^^12,13^. Based on an average weight of an adult in the US of 84 kg, this would be equivalent to 5.9 mg of NO_2_^-^ or 310.8 mg of NO_3_^-^ in an average adult weighing 84 kg. The American Cancer Society guideline for diet and physical activity for cancer prevention also recommends limiting red meat and processed meat consumption^14^. However, the concentration of NO_2_^-^ and NO_3_^-^ can vary by class of processed meats, suggesting health implications may differ by type of processed meat.

N-nitrosamine formation is generally considered to occur by the reaction of residual NO_2_^-^ in processed meat and biogenic amines in the acidic environment of the gastrointestinal tract^15^. Thus, processed meat products with higher residual NO_2_^-^ could present higher health risks. Processed white meat such as cured whole poultry products generally possess higher residual NO_2_^-^ than processed meats made from red meats since they would contain less myoglobin to react with the NO_2_^-16^. Residual NO_2_^-^ is minimal in fermented and acidified processed meats but dietary nitrosamine may form in those products before consumption^17,18^. Understanding the amount of NO_2_^-^ and NO_3_^-^ in processed meats and how processing affects their concentration may help guide consumers’ dietary choices and grocery selections.

Furthermore, color is one of the most important characteristics that consumers use to assess meat quality and freshness^19^. There is a limited understanding of how NO_x_^-^ content impacts color attributes and their correlation during storage. Cured meat color may develop initially and then fade to a paler color over the course of storage, particularly under lighted display or if oxygen has not been completely excluded from the package. Residual NO_x_^-^ serves as a reservoir to re-generate cured meat pigments, and thus generally correlates with cured meats color^20^. The color of fermented, acidified and dehydrated meats generally appears redder and more palatable due to the rapid utilization and consequently depletion of NO_2_^-^ during processing. Previous studies collected products from either groceries stores or unstandardized manufactory time that had considerable variation in proximity to their *best by* dates, and as such limited the ability to determine how nitrite levels decline over storage time and its relationship to changes in color stability^2,21^.

Previous studies have reported the amount of residual NO_2_^-^ and NO_3_^-^ in processed meats from different regions of the United States^2,21,22^. However, there is a lack of systematic studies on the (1) depletion of NO_2_^-^ and NO_3_^-^ in commercial processed meats during the course of storage (as the previous studies analyze product without a standardized manufactory time frame); (2) effects of formulation, processing, and meat species on NO_x_^-^ content in processed meats; (3) NO_x_^-^ content and its correlation with product color during storage; and (4) NO_x_^-^ content in newly-evolved plant protein-based meat analogues. Therefore, the objectives of this study were to (1) evaluate the NO_x_^-^ content of products from various major categories of cured and processed meats manufactured by small and regional meat processors across three geographic locations; (2) categorize variations of residual NO_x_^-^ in these products based on species of meat, addition of non-meat ingredients, and processing methods ; (3) conduct a depletion study in these products to understand correlation of residual NO_x_^-^and color during the course of storage using meat products from regional meat association competitions; and (4) assess the residual NO_x_^-^ content of plant protein-based meat analogues.

## Results and discussion

### NO_x_^-^ in major classes of processed meats

**Fig. 1:**
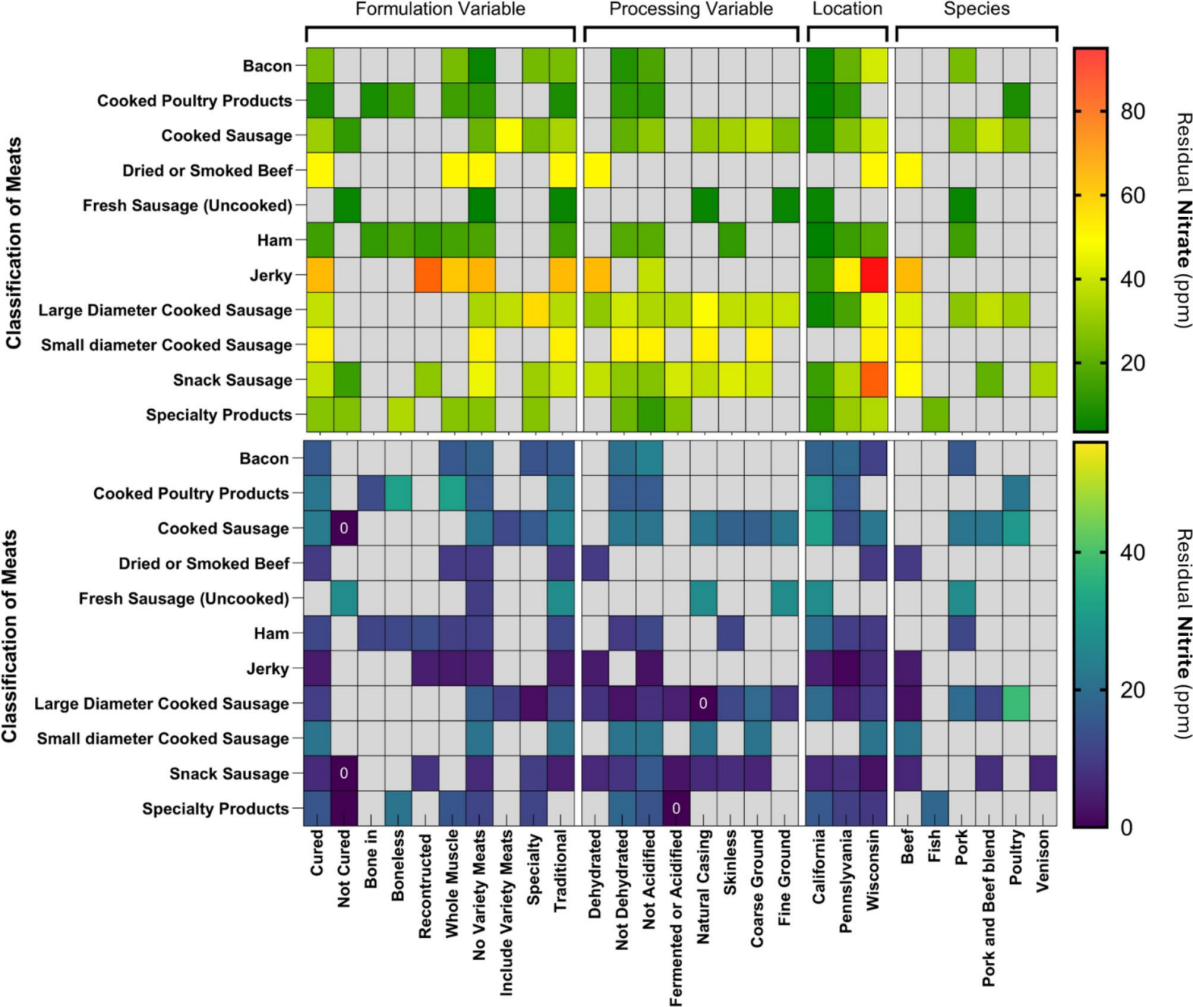
Major classes of processed meats from three different geographic regions (California, Wisconsin, and Pennsylvania) in the United States (*n* = 1106). Grids in gray color indicate data not applicable; Grids denoted 0 indicate residual NO_x_^−^ below the detection limit.

A total of 1132 uncooked processed meats, and cooked whole muscle cured meats, and comminuted cured meats were collected during State meat product competitions organized by the meat processors associations of California (*n* = 220), Pennsylvania (*n* = 301), and Wisconsin (*n* = 611; 469 in 2023, 132 in 2024) (Table 1). The residual NO_x_^−^ content of thirty-two classes of processed meat samples collected at the 2023 Wisconsin Association of Meat Processors (WAMP) product competition generally did not differ significantly (*p* < 0.05) from that of samples collected in 2024 at the same competition. The residual NO_2_^−^ and NO_3_^−^ content in all classes of processed meat products, and how processing methods, formulation variables, species of meat, and geographic location correlated with them, are shown in Fig. 1. The mean residual NO_2_^−^ content (in ppm) in decreasing order was: fresh (uncooked) pork sausage (26.9) > cooked poultry products (21.9) > cooked sausage products (21.7) > small diameter cooked sausage (10.2) > bacon (15.3) > ham (11.7) > specialty products (11.3) > large diameter cooked sausage (10.1) > dried smoked beef (9.5) > snack sausage (6.3) > jerky (3.7). The mean residual NO_3_^−^ content (in ppm) in decreasing order was: jerky (61.3) > dried or smoked > small diameter cooked sausage (51.6) > beef (50.2) > large diameter cooked sausage (38.1) > snack sausage (31.3) > cooked sausage products (30.5) > specialty cooked meats (27.2) > bacon (24.8) > ham (14.3) > cooked poultry products (8.2) > fresh pork sausage (5.0). These data generally show a similar trend of NO_2_^−^ and NO_3_^−^ content across the different classes of processed meats, as do previous studies^2,21,23^, . NO_2_^−^ content was lowest in snack sausage and jerky-type products whereas NO_3_^−^ content was lowest in cooked poultry products and uncooked pork sausage. In the same region of the country, uncooked raw pork sausage contains residual NO_2_^−^, possibly added to improve color appearance during retail display^2,24^. This practice is particularly useful for vacuum-packaged meat products, since nitrosometmyoglobin will be reduced to nitrosomyoglobin under anaerobic conditions, leading to a bright red color^25^.

**Table 1:** Total number of cured and processed Meat products Collected during State Meat associations Product conventions in the United States.

Product category	Product subcategory	Collecting location and year			
		California	Pennsylvania	Wisconsin	
		2024	2023	2023	2024
Cured cooked sausage	Cooked sausage, large diameter sausage, small diameter cooked sausages	60	84	290	24
Fermented and acidified sausage	Snack sausage, summer sausage	43	87	43	26
Whole muscle brine cured	Bacon, cooked whole poultry products, bone-in and boneless ham	66	49	87	27
Cured and dehydrated	Jerky, dried or smoked beef	15	71	42	30
Fresh Sausage	Fresh sausage, fresh pork sausage	36	10	16	26
Total		220	301	478	133

3.2 Inferences postulated associated with geographic location, meat species, formulation variables, and processing methods on residual NO_x_^-^ in processed meats.

NO_x_^−^ in processed meat products are primarily introduced by the addition of added nitrite and nitrate as curing ingredients. Nitrites are commonly used as curing ingredients in most cured meat products because of their quick-acting properties. In contrast, a limited number of products, such as dry-cured ham and dry-cured salami, use nitrate as a reservoir for nitrite generation within the meat through the action of the microbial enzyme nitrate reductase^26,27^. Compared with many other countries in the world that regulate the NO_x_^−^ content of finished products^28^, the United States Department of Agriculture regulates the maximum ingoing (added) nitrite or nitrate in meat products. These amounts, for sodium nitrite and sodium nitrate, are 200 and 700 ppm for whole muscle products and 156 and 1718 ppm for comminuted products, respectively^8^.

**Fig. 2:**
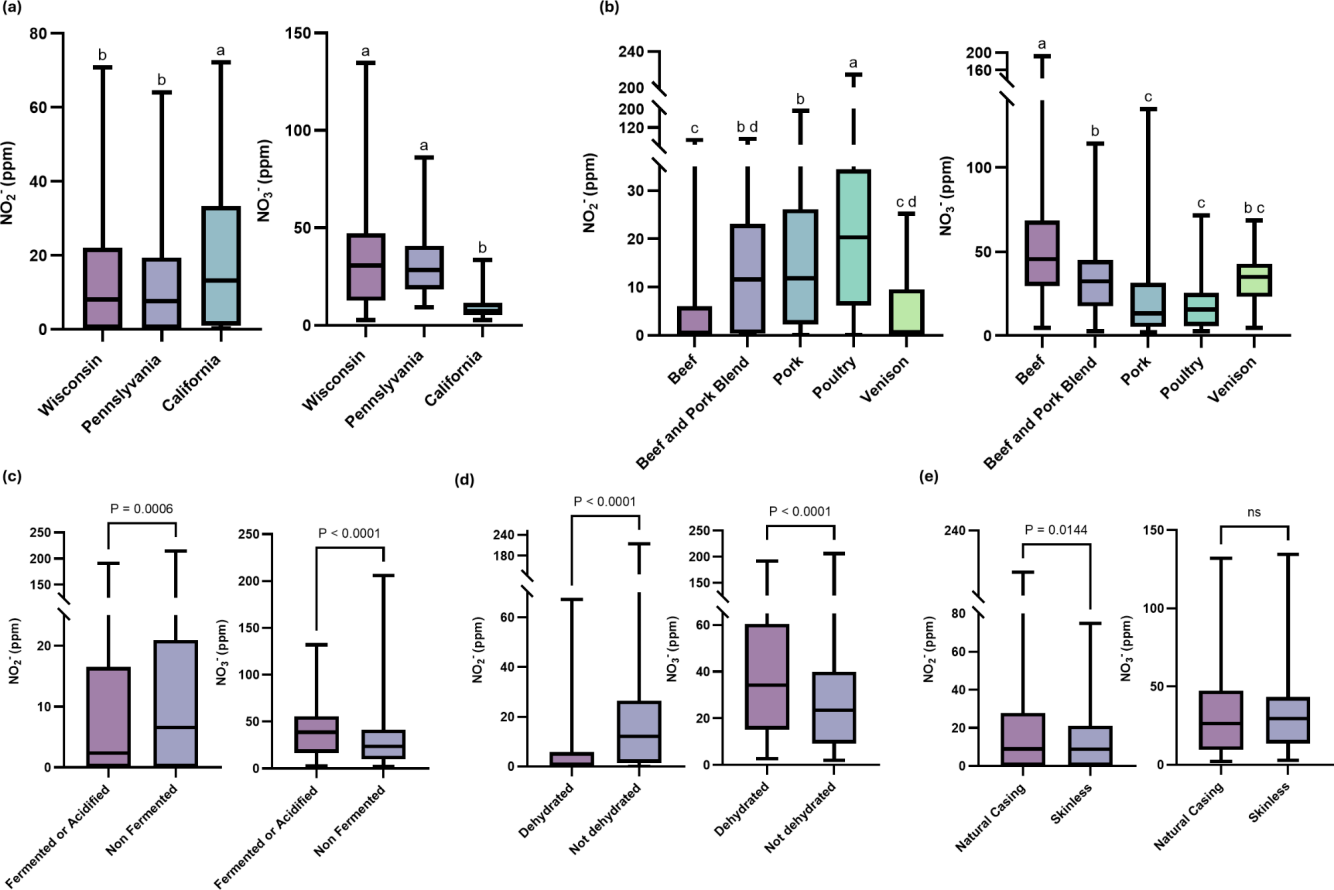
Effects of geographic location, species of meat, processing, and formulation variables on residual NO_2_^−^ and NO_3_^−^ in all major classes of processed (*n* = 1132). (**a**) Effects of geographic location on residual NO_x_^−^ in processed meats (*n* = 963). (**b**) Effects of species of meats (**c**) Effects of fermentation and acidification on residual NO_x_^−^ in processed meats. (**d**) Effects of dehydration on residual NO_x_^−^ in processed meats. (**e**) Effects of casing on residual NO_x_^−^ in processed meats. Different letters within figure a and b, and specific NOx- are different (*p* < 0.05) In figures c-e, P-values denotes significant difference between paired comparisons.

The NO_2_^−^ content of processed meats collected in California, Pennsylvania, and Wisconsin averaged (± standard error; minimum and maximum values in parentheses) 19.4 ± 1.9 (0.2–71.9), 11.3 ± 1.4 (0.0–64.1), and 14.0 ± 1.2 (0.0-70.8) ppm, and the NO_3_^−^ content averaged 9.3 ± 0.6 (2.7–33.6), 31.7 ± 1.7 (9.3–86.2), and 34.5 ± 1.3 (2.7-134.6) ppm, respectively (Fig. 2a). Processed meat samples collected during the California regional product competition were significantly higher (*p* < 0.0001) residual NO_2_^−^ than those from the Wisconsin and Pennsylvania competitions. The higher NO_2_^-^ content was contributed by residual NO_2_^-^ in uncooked pork, poultry and regular sausage products from California (NO_2_^-^ content in uncooked sausage measured in the raw product). Residual NO_2_^-^ content in uncooked sausage samples has a much slower depletion rate than in other processed meats that undergo thermal processing, which is known to accelerate NO_2_^-^ conversion and depletion^29^. On the other hand, residual NO_3_^−^ content was significantly different among Wisconsin, Pennsylvania and California processed meat samples. In Wisconsin, potable water NO_3_^-^ content from product manufacturing locations were evaluated and show little to no correlation (*r* < 0.25) with processed meat products manufactured therein^30^. Geographic differences in NO_3_^-^ could have resulted from differences in spice blend usage and formulations (specialty formulation that incorporates more spices, cheese and vegetables versus traditional formulation) in different regions of the United States, as all other variables (water, meat ingredients, and formulation aids) showed little to no correlation with residual NO_3_^-31-33^.

The species of meat used in the processed meat affected residual NO_2_^-^ and NO_3_^-^ content (*p* < 0.05) (Fig. 2b). Processed meats made of beef contained the lowest residual NO_2_^-^ content and the highest NO_3_^-^ content, driven by the wide use of beef in snack sticks and dehydrated beef jerky. A higher level of NO_2_^-^ was observed in poultry products than in the other meat species, which agrees with previous reports^16^. This is possibly due to the lower abundance of heme in poultry meat compared to red meat, where the heme content facilitates the conversion of nitrite (NO_2_^-^)^34^. The increased NO_2_^-^could improve the texture profile of processed meat products by decreasing α-helix and β-sheet conversion and reducing myofibrillar protein oxidation^2,6^. Processed venison products are typically made into dehydrated meat snacks or jerky in the United States, resulting in a similar nitrate (NO_3_^-^) content as beef^35^.

Fermentation or acidification reduces NO_2_^-^ (*p* = 0.0006) and increases NO_3_^-^ content (*p* < 0.0001) in processed meats (Fig. 2c), while dehydration similarly lowers NO_2_^-^ (*p* < 0.0001) but raises NO_3_^-^ content (*p* < 0.0001) (Fig. 2d). Fermentation and acidification converts more reactive NO_2_^-^ to nitric oxide during fermentation and thermal treatment^36^, while the dehydration process used in the production of snack sticks and jerky generally concentrates NO_3_^-^, which is generally more stable at room temperature^37^.

Natural casing frankfurters had higher NO_2_^-^ (*p* = 0.0144) than skinless frankfurter products, whereas NO_3_^-^ was not different between these two types of sausages (Fig. 2e). This difference may potentially be simply due to formulation decisions by the meat processors^16,38^. The results of Fig. 1 suggest that residual NO_2_^-^ is higher in boneless products than in bone-in products, possibly due to more contact surface of lean meat to brine in boneless products than that of bone-in injected products. Furthermore, reconstructed beef jerky and whole muscle jerky did not differ in residual NO_x_^-^ content. These observations indicate that formulation and processing influence residual NO_x_^-^ content in processed meats.

### Depletion study on residual NO_2_^-^ and NO_3_^-^ and their correlation with color in processed meats

Color is considered one of the most important sensory attributes of processed meat products that consumers rely on to evaluate meat quality and freshness^39^. The color stability and residual nitrite depletion study were applicable due to the meat products collected having been manufactured within a week of submission to their respective state processed meat competitions, as opposed to products from retail stores, which may be considerably older and of varying age. During the meat curing process, added NO_2_^-^ and NO_3_^-^ are reduced to nitrite oxide by Fe^2+^ from myoglobin, NADH or NADPH associated with the TCA cycle, or by added curing accelerators, such as sodium erythorbate, and natural cure accelerators such as cherry or acerola powder, which have abundant amounts of ascorbic acid^40–42^. Nitric oxide can then react with the raw meat pigment forms metmyoglobin or deoxymyoglobin (oxidized and reduced heme iron state, respectively) to form nitrosometmyoglobin or nitrosomyoglobin, respectively. Subsequent thermal processing denatures these pigments and creates reducing conditions to form nitrosohemochrome, the stable pink pigment responsible for the characteristic pinkish color of cooked cured meat and poultry products^43–46^. However, this cooked cured meat pigment is susceptible to photooxidation and oxidation associated with oxygen exposure, thus requiring most processed meats to be preserved by vacuum packaging and proper light display^47^. Residual NO_2_^-^ and NO_3_^-^ also play a role in color preservation of cured meat products, since they can interconvert to each other or nitric oxide, and can thus serve as a “reservoir” to help preserve the pink color of cured meat pigments^48^. These properties of cured meats warrant a time-dependent study of color change that can help consumers understand how color correlates with the freshness of cured meats.

**Fig. 3:**
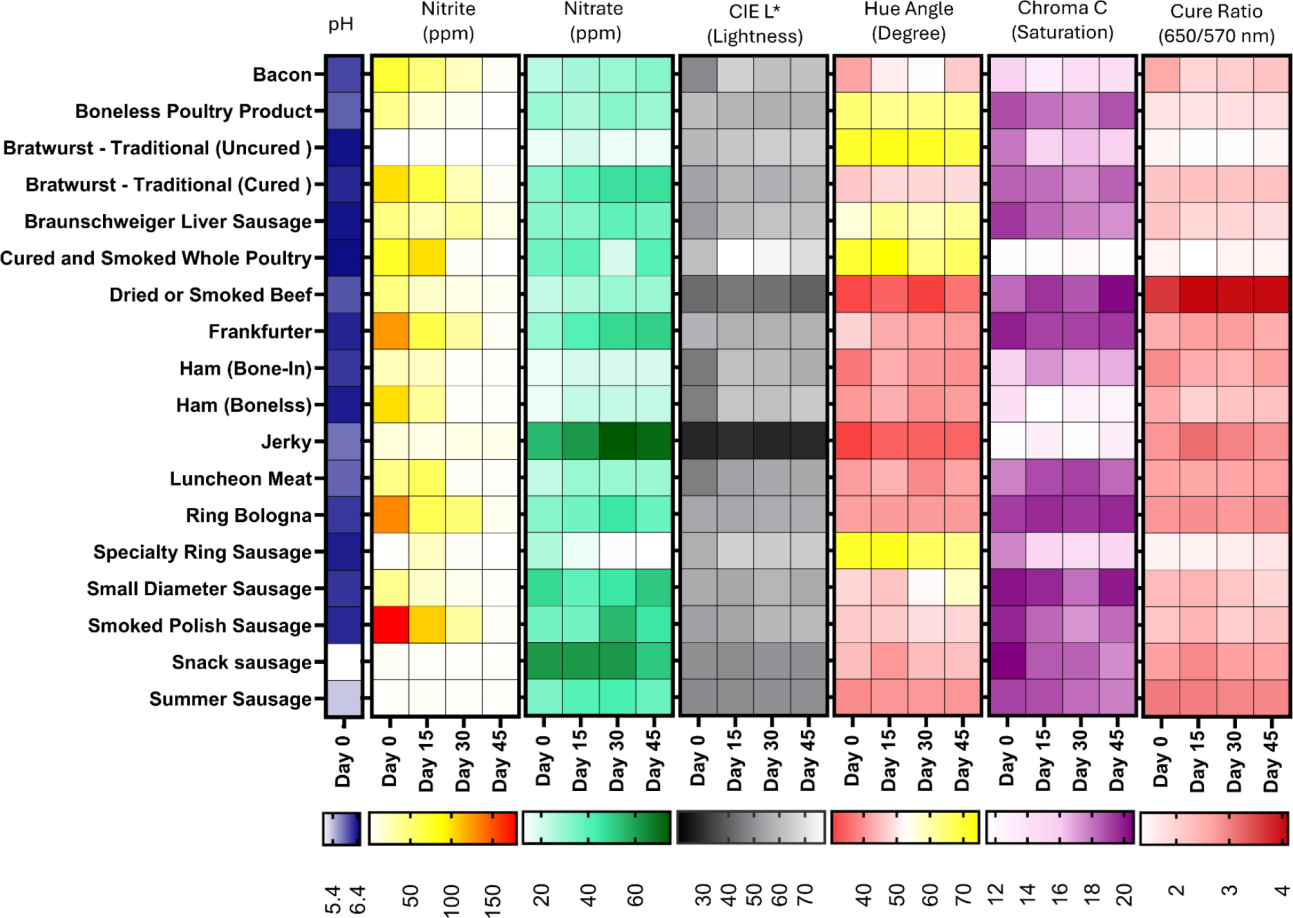
Depletion study of residual NO_2_^−^ and NO_3_^−^ and visible color attributes and cure ratio in all major classes of processed meats in the United States (*n* = 113). Day 0 in this study represents the earliest time products can be available to consumers. Nitrosohemochorme (cured meat pink pigment) determined as a reflectance ratio (% reflectance at 650 nm / % reflectance at 570 nm).

Figure 3 shows the change in color as measured by CIE L* (lightness), hue angle (primarily in the red to yellow region), Chroma C (color saturation), and cure color ratio (spectrophotometric determination of % reflectance at 650 nm/570nm, predictor of nitrosohemochrome presence) and their correlation with residual NO_x_^−^ over 45 days of refrigerated display storage. Generally, NO_2_^−^ progressively decreased to a very low content, whereas NO_3_^−^ content was stable or increased slightly over the course of the storage period. The increase in NO_3_^−^ likely was associated with the oxidation of some of the residual nitrite during storage. The color in many of the classes of cured meats faded slightly to a paler color (as indicated by higher CIE L* and lower chroma c) while the color of fermented or dehydrated cured meats remained constant during storage. In some cured meat classes, such as jerky, cured bratwurst, frankfurter, and snack sausage, cured color, measured by the cure color ratio, improved slightly on day 15 and decreased thereafter. The redness or yellowness of the products, indicated by the hue angle, was relatively stable during storage.

**Fig. 4:**
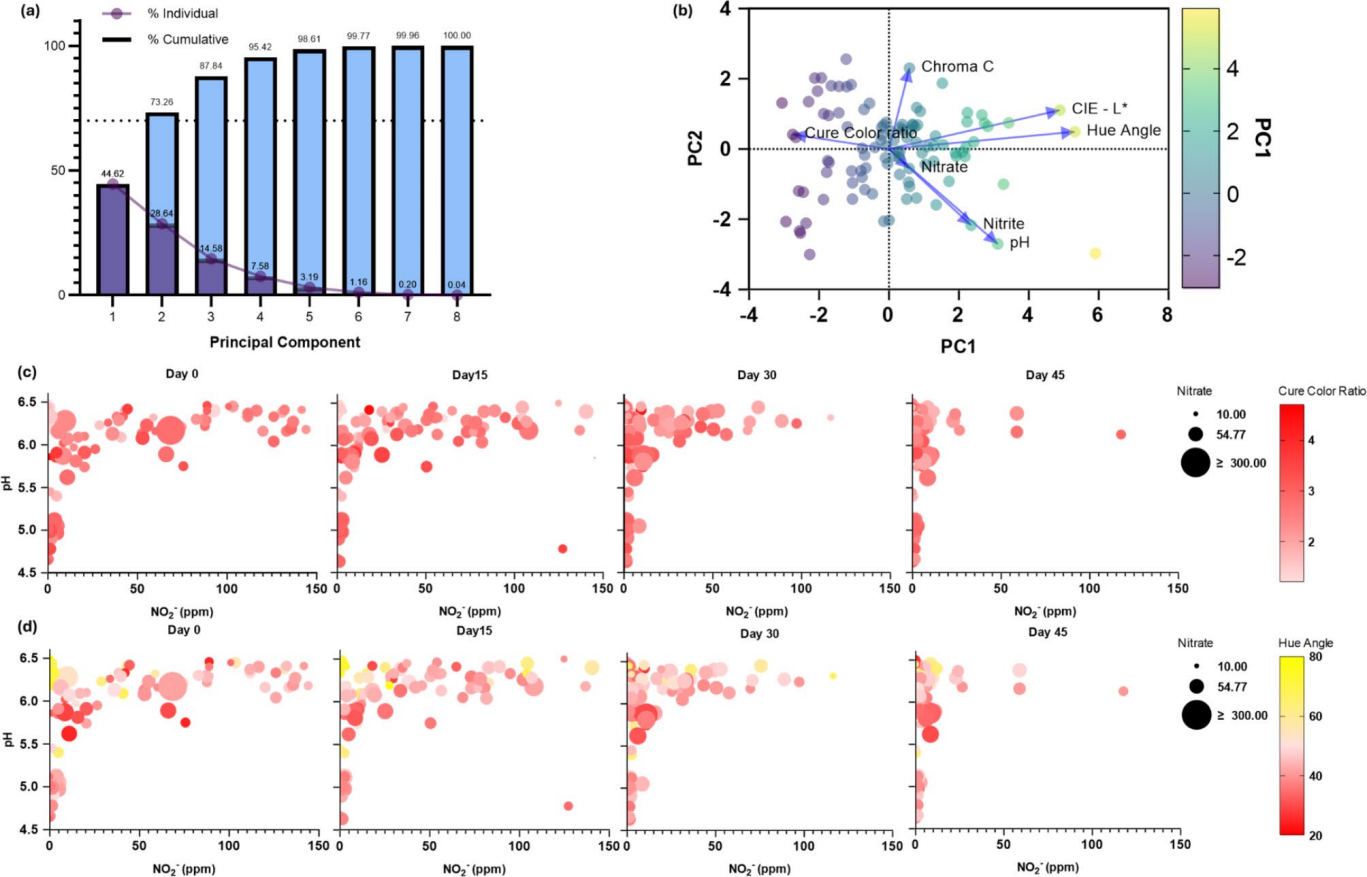
Principal Component Analysis (PCA) on cured meats color and residual NO_2_^−^. (**a**) Proportion of variance indicates applicability of PCA analysis; (**b**) Biplot of cured meat color to NO_2_^−^; (**c**) Correlation of residual NO_x_^−^ with cure color ratio at (reflectance: %R 650 nm/%R 570 nm) during 45 days of storage; (**d**) Correlation of residual NO_2_^−^ with hue angle during 45 days of storage. PCA was done using data from Day 0 products from Wisconsin (*n* = 113).

Principal Component Analysis (PCA) indicated that NO_x_^−^ concentration had a strong positive correlation with pH and a weak positive correlation with Hue Angle (Fig. 4). CIE L* had a strong negative correlation with cure color ratio during storage. The higher amount of NO_x_^−^ is likely related to a distinctive pinkish cured meat color and likely served as a “reservoir” to help stabilize the cured pigment in unacidified or non-fermented products. Interestingly, acidified or fermented meat products which had limited residual NO_2_^−^, displayed excellent color over 45 days of display storage. In acidified cured meats, most of the NO_2_^−^ content is rapidly converted to nitrous acid and reacts at a higher rate with other substances such as proteins and amino acids during the initial curing and fermentation process before thermal treatment. During fermentation or chemical acidification processes, nitric oxide reacts with myoglobin and metmyoglobin, possibly producing forms of nitrosometmyoglobin that may be structurally different from those of the regular curing process and lead to a more stable pink color during storage^16,49,50^. Higher acidity tends to lead to a better cure color in terms of hue angle and cure color ratio. However, rapid protonation of nitric oxide to nitrous acid in a high acidic condition may increase the reaction rate of nitrosamine formation; thus the pH of meat acidifying processes should be closely examined^36^.

### Residual NO_2_^-^ and NO_3_^-^ in plant-based meat analogues

Residual NO_x_^−^ content in plant protein-based meat analogues is an under-investigated area^51^. However NO_x_^−^ may be introduced into meat analogues by functional binding and gelling agents originating from plant materials, such as soy, pea and wheat protein, and ingredients in flavoring sauces, such as apple and pear purees^52,53^. N_2,_ NO, and NO_2_^−^are key nutrients that support plant growth through their involvement in different metabolic mechanisms and, as such, are eventually assimilated at different levels in harvested fruit and vegetable products destined for human food and food ingredients^54–56^. There are currently no regulations limiting the amount of residual NO_2_^−^ and NO_3_^−^ in plant-based meat analogues, unlike conventional animal protein meat products where added NO_x_^-^ are regulated based on their ingoing concentration in the U.S^52,57,58^.

**Fig. 5:**
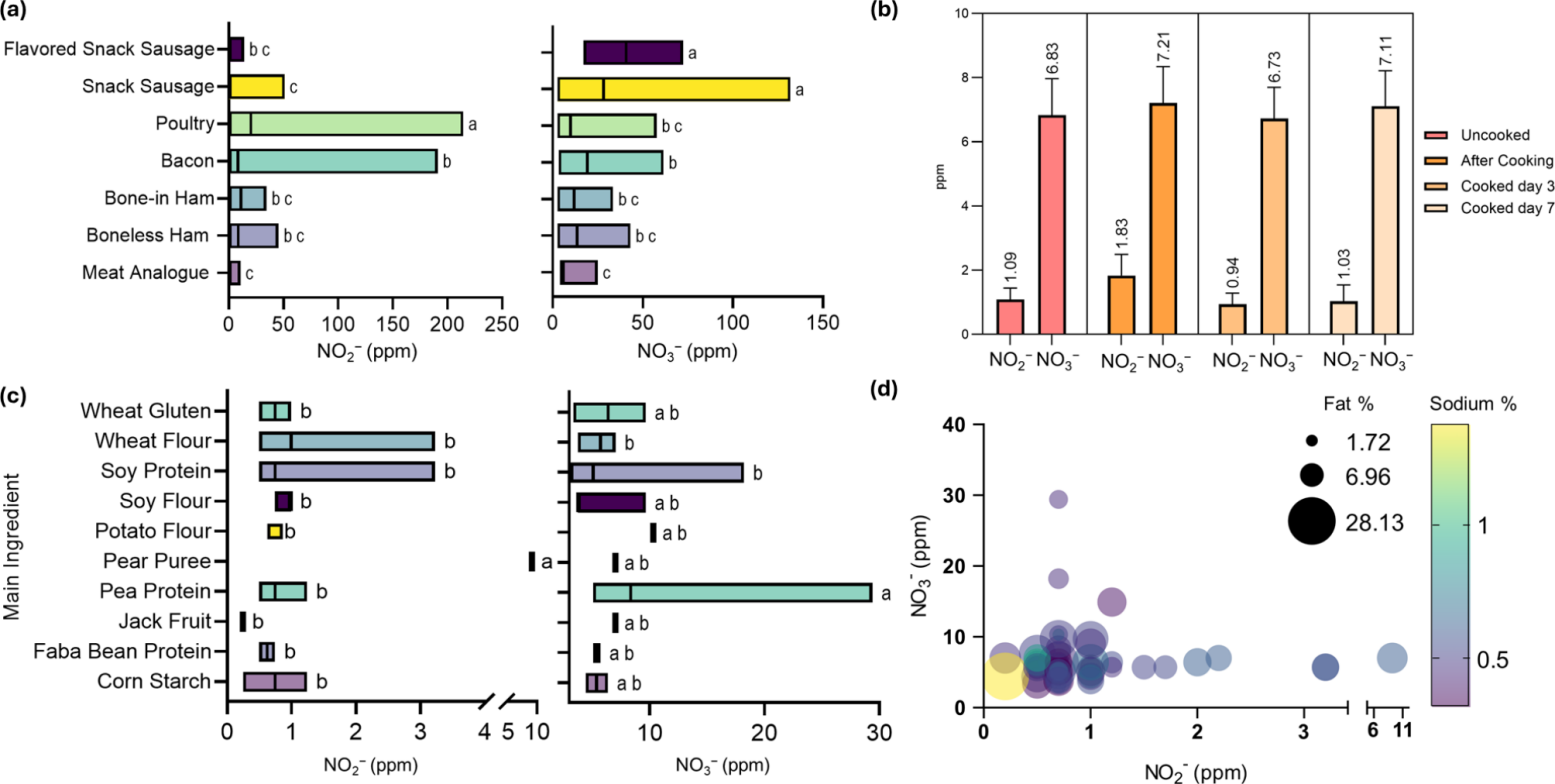
Residual NO_2_^−^ and NO_3_^−^ data for plant-based meat analogues (*n* = 53). (**a**) NO_x_^−^ in plant-based meat analogues (*n* = 53) comparison with common animal protein cured meat products; (**b**) NO_x_^−^ in plant-based meat analogues in package, after cooking, day 3, and day 7 days stored in refrigerator (temperature = 34.2 °F); (**c**) NO_x_^−^ in plant-based meat analogues (*n* = 53) based on main plant protein ingredients; (**d**) bubble chart for residual NO_2_^−^ and NO_3_^−^ in plant-based meat analogues. Size of bubbles indicate total fat percentage from 1.72 to 28.13%. Color of bubbles indicate sodium percentage from 0.35 to 1.47%. Unlike letters denote difference (*p* < 0.05).

As shown in Fig. 5, meat analogues contain similar levels of NO_2_^−^ as common, conventional, cured processed meats such as snack sausage and ham products (*p* > 0.05), and similar levels of NO_3_^−^ as cured whole or processes poultry products and bone-in or boneless ham (*p* > 0.05). The NO_2_^−^ and NO_3_^−^ content in meat analogues averaged (± standard error, minimum and maximum values in parentheses) 1.66 ± 0.34 (0.00–11.00) and 7.17 ± 0.56 (4.00-25.25) ppm, respectively.

The ingredient statement analysis of plant protein-based meat analogues indicated that pear puree containing products have the highest NO_2_^−^ content (*p* < 0.05) and the pea protein containing products have the highest NO_3_^−^ content (*p* < 0.05). Soy and wheat protein-containing meat analogues had a mean NO_3_^-^ (± standard error, minimum and maximum values in parentheses) content of 5.7 ± 1.9 (3.8–9.7) and 6.1 ± 0.7 (3.1–18.2) ppm, respectively. The correlation study (results not shown) conducted using Pearson’s correlation indicated a weak correlation between NO_x_^-^ concentration and other nutritional factors such as total fat, saturated fat, protein, and sodium content. NO_3_^-^ content had a slight positive correlation with pH level (*r* = 0.26). Sodium content had a positive correlation with total fat percent (*r* = 0.65).

The overall results of this study show that some plant-based meat analogues contain similar NO_x_^−^ content as some common processed meats. The NO_2_^−^ and NO_3_^−^ content of plant-based meat analogues do not appear to be affected by cooking and storage (*p* < 0.05) and deplete much slower than conventional cured and processed meat products. Further investigation of possible reactions of NO_x_^−^ with other substances in plant-based meat analogues subjected to different cooking, processing, storage and handling conditions is warranted.

### Limitations of this study

The study on processed meats utilized meat samples collected from three state meat association annual product competitions and may have excluded alternatively-cured (i.e., vegetable powder-cured) meat products. Most sampled products were cured by direct addition of sodium nitrite, as opposed to NO_3_^–^ or NO_2_^–^-containing vegetable powders, which may provide different quality attributes to processed meats. Therefore, further research on NO_x_^−^ content and quality attributes in all major classes of alternatively-cured processed meat products is warranted to complement this study. This study used an observational approach similar to a previous nationwide study on residual NO_x_^−^ in processed meats^2^. Future studies on the residual NO_x_^−^ content of processed meats and meat analogues are warranted.

## Conclusion

Pre-thermal processing conditions such as ingoing nitrite level, acidifying, fermentation and formulation (traditional formulation versus specialty formulation) play an important role in impacting NO_x_^−^ content in processed meats. In non-acidified and unfermented processed meats, the presence of residual NO_2_^−^ likely contributes to maintaining a desirable color and color stability. In contrast, in acidified, fermented, and dehydrated processed meat quality attributes (color and appearance) appear to be independent of residual NO_x_^−^. Plant protein-based meat analogues may contain a certain amount of NO_2_^−^ and NO_3_^−^ introduced from plant ingredients which are akin to some common processed meat products. The concentration of NO_x_^−^ depends on the ingredients and pH of each individual meat analogue product. The results of this research provide an updated baseline for the major categories of conventional processed meats and meat analogue in different regions in the United States and present consumers a comprehensive, time-dependent study on NO_x_^−^ depletion in the major classes of processed meats, while providing information on the correlation of NO_x_^−^ and color attributes.

## Materials and methods

### Sample selection and collection

Samples of cured and processed meat samples (*n* = 1,185) were collected during the California, Pennsylvania, and Wisconsin meat processor annual product competitions, held between April 2023 and April 2024. Sample collection in Wisconsin was conducted both in 2023 and 2024. Processors entering the Wisconsin and California competitions were restricted to local processors only. Processors entering the Pennsylvania Association of Meat Processors product competition were from all New England area states. Samples of meat analogues (*n* = 53) were purchased from local grocery stores in Madison, Wisconsin. Samples collected in Wisconsin were stored in insulated coolers lined closely with icepacks and transported to the Meat Science and Animal Biologics Discovery (MSABD) building at the University of Wisconsin-Madison within an hour after collection. Samples collected in Pennsylvania and California were frozen before being placed in a cooler lined with frozen ice packs (covering the bottom, sides, and top) and transported to the MSABD’s freezer within the same day. The entire shipping process took less than 12 h, with the samples kept in cooler boxes packed with ice packs. All samples were vacuum packed at -77,500 Pa using a commercial meat vacuum sealer (Vacmaster VP215, Vacmaster Co., Greenville, SC, U.S.A.) in a lab illuminated by LED lights that do not emit UV light, upon delivery to MSABD. They were then immediately stored in a dark environment at -20 °C. All analyses were completed within 5 days after receipt of the samples. Sample of 53 plant protein-based meat analogue products were purchased in store in Madison, WI and held frozen (-20 °C) until analysis. Meat analogue products were cooked following manufacturer recommended cooking instructions on day 0, day 3, and day 7 and analyzed for residual NO_2_^−^ and NO_3_^−^content. Day 3 and Day 7 cooked products were stored individually within a Styrofoam tray wrapped with oxygen permeable film (AEP Industries Inc., South Hackensack, N.J. U.S.A. Model: Sealwrap with oxygen transmission rate at 98.4 cm^3^/100 cm^2^/day and water vapor transmission rate at 621 g/m^2^/day at 37.8 °C with 100% Relative Humidity) in a temperature controlled (3°C) dark environment before analysis.

### Depletion study setup

One hundred and twenty-six (126) samples (statistical power = 0.81) were randomly selected and collected from the Wisconsin Association of Meat Processors (WAMP) annual product competition held in Middleton, WI in April 2024. Each sample was divided into four vacuum-sealed polyethylene bags containing approximately 100 g of comminuted products or at least two slices of whole muscle products representing sample’s overall lean and fat ratio (e.g. a center slice of a bacon representing the overall lean and fat ratio of a bacon slab) and stored in a temperature controlled (3 °C) dark environment prior to analysis (pH, residual NO_x_^−^, and color) on days 0, 15, 30, and 45, following the methods described below.

### Nitrite and nitrate content determination

**Fig. 6:**
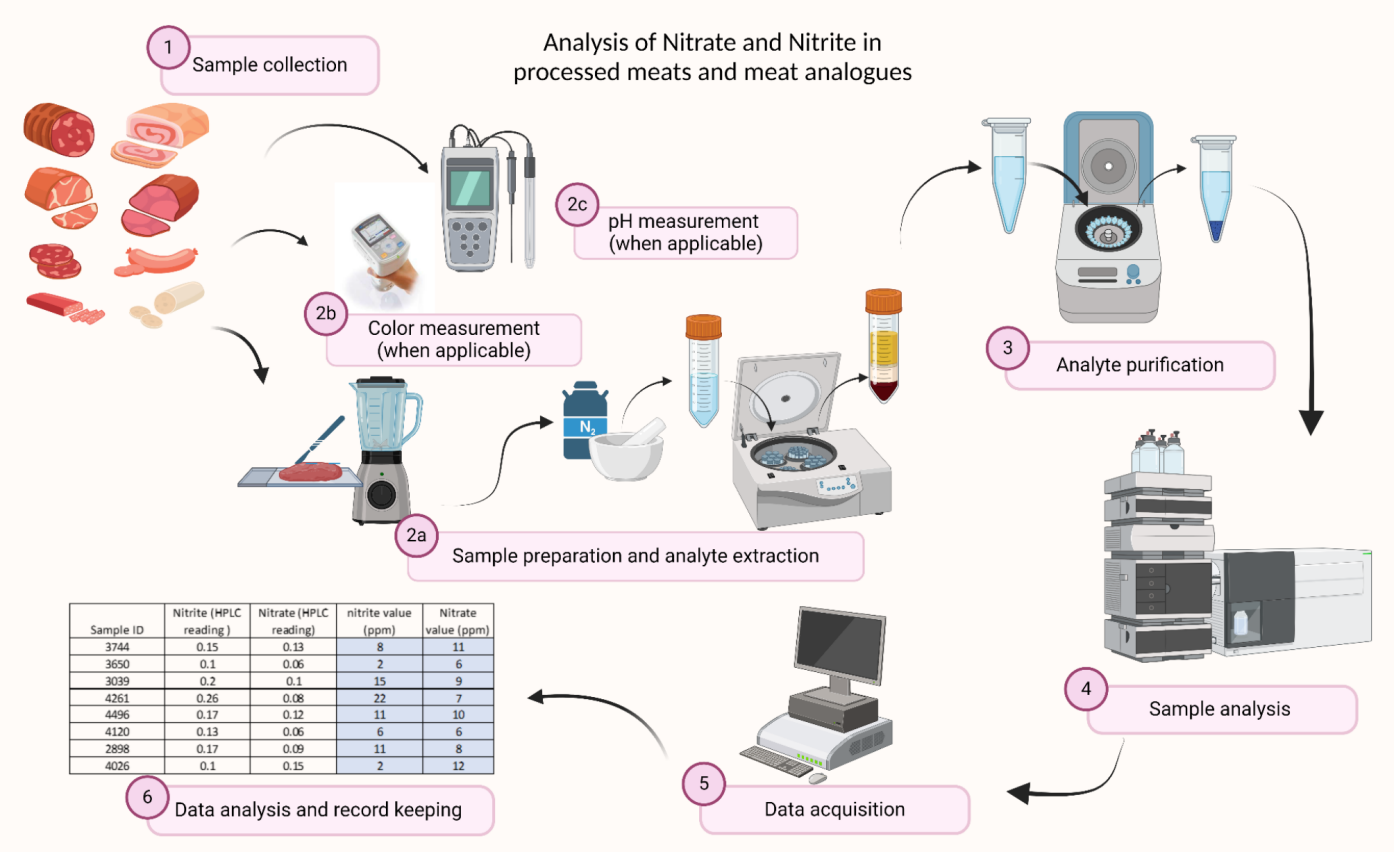
Scheme of residual NO_2_^−^ and NO_3_^−^ determination and quality measurements (color and pH) in processed meats and meat analogues (Created with BioRender.com).

Residual NO_2_^−^ and NO_3_^−^ were analyzed using a high performance liquid chromatography (HPLC) equipment (ENO-20 NO_x_ Analyzer, Eicom Inc, Kyoto, Japan) coupled with a temperature controlled autosampler (AS-700, Amuza Inc., San Diego, C.A., U.S.A.) according to the method described by De González et al., with modifications^2^ (Fig. 6). The HPLC analysis for NO_x_^-^ was designed based on the Griess nitrite test adopted by the Association of Official Analytical Chemists (A.O.A.C)^59,60^. A UV-Vis detector was built into the HPLC to detect absorption at 540 nm. A reverse-phase separation column separates nitrites and nitrates in the analyte. Then, a reduction column coated with cadmium reduces nitrates to nitrites. The nitrites then react with sulfanilamide and N-1-naphthylethylenediamine dihydrochloride in an acidic environment, forming a pinkish diazo dye that has an absorbance at 540 nm. Samples (processed meats and meat analogues) were powdered in liquid nitrogen and stored at − 80 °C until analysis. A 5-gram sample was weighed into 45 mL of pH 7.4 phosphate-buffered saline (PBS) and then split into two equal-volume slurries and centrifuged at 3,500 × g at 4 °C for 5 min (J6-MI centrifuge equipped with JA-25.50 rotor; Beckman Coulter, Indianapolis, IN, U.S.A.). After centrifugation, 500 µl of supernatant from each slurry and 500 µl of 100% methanol were mixed, transferred to a 1.5-mL snap-cap centrifuge tube, and vortexed for 10 sat 3000 rpm with a digital vortex mixer (cat. no. 0215370, Fisher Scientific, Hanover Park, IL U.S.A). The samples were then centrifuged for 16 min at 15,000 × g at 4 °C (Eppendorf 5424 centrifuge, Brinkmann Instruments, Westburg, NY, U.S.A.). Two hundred-µL supernatants were pipetted into 96-well plates for quantification with the HPLC equipment described above. Quantitative data (area under the curve) were analyzed with PowerChrom (version 16.0, New South Wales, Australia). HPLC carrier pump speed was set at 40 ml/hour and reactor pump speed was set at 13.2 ml/hour. A calibration curve was created using 2, 4, 8, 16 ppm of sodium nitrite and sodium nitrate. A sodium nitrite standard (8 ppm) was tested at the start and end of each run on a daily basis.

### Color measurement

Color measurement was conducted according to the Guidelines for Meat Color Measurement of the American Meat Science Association (AMSA) using a Konica Minolta CM-600D spectrophotometer (Konica Minolta Sensing Inc., Osaka, Japan). The device was equipped with illuminant D65, an 8 mm aperture, and a 10° standard observer. Data collected were averaged for statistical analysis^61^. A minimum of ten scans were conducted on each sample of processed meats samples, outliers (z-score above 3 or below − 3) were excluded from calculation. Processed meat color was measured through oxygen-permeable film (AEP Industries Inc., South Hackensack, N.J. U.S.A. Model: Sealwrap with oxygen transmission rate 98.4 cc/100 cm^2^/day) after calibrating the spectrophotometer with the accompanied standard white plate covered with oxygen-permeable firm placed over the tile (SM-A177, No. 12671). The CIE L*, a* and b* readings and reflectance values from 450 to 700 nm at 10 nm intervals were used to characterize cured color (Hue angle is calculated as h = arctangent (b*/a*), Chroma (saturation index) calculated as (a*^2^ + b*^2^)^0.5^)^61^.

### pH measurement

pH of samples was determined by blending the samples with ultra-pure water (resistivity of 18.2 MΩ.cm) at a 1:9 ratio using a polytron blender at 15,000 Revolutions per minute(rpm), then measuring with a pH meter (Fisherbrand™ Accumet™ AE150; Fisher Scientific Inc., MA U.S.A.) equipped with an AE Series 3-in-1 Single Junction Gel pH/ATC electrode (Fisherbrand™ Accumet™ model 13-620-AE6; Fisher Scientific Waltham, MA, U.S.A.). Calibration of the pH meter was conducted with NIST certified potassium biophthalate buffer (pH = 4.00 ) and potassium monobasic and sodium hydroxide buffer (pH = 7.0) following the procedure described by Neto et al.^62^. The pH meter was calibrated daily and measurements were conducted in duplicate.

### Data collection and statistical analysis

HPLC data were collected using chromatography data collection software (Powerchrome 16.0). A multi-linear regression model was applied to evaluate factors that contributed to residual nitrite and nitrate level in processed meats; the D’Agostino-Pearson test was conducted to assess normality between samples. One-way ANOVA test and pairwise t-tests were used to assess statistical differences between multiple samples. P values of < 0.05 were considered statistically significant. Pearson r tests were conducted to test whether relationships between variables were significant. All statistical analysis were performed using R, version 4.3.3 (www.r-project.org).

## Electronic supplementary material

Below is the link to the electronic supplementary material.

Supplementary Material 1

## Data Availability

The datasets generated and/or analyzed during the current study are available from the corresponding author upon reasonable request.

## Abbreviations

(NO_2_^-^): Nitrite
(NO_3_^-^): Nitrate
(NO): Nitric oxide
(HPLC): High performance liquid chromatography
(CIE): International Commission on Illumination
