# Supplementary material for: Residual nitrite and nitrate in processed meats and meat analogues in the United States
Source: Sci Rep. 2025 Jan 25;15:3269. doi: 10.1038/s41598-025-87563-x (PMC11763100; doi:10.1038/s41598-025-87563-x)
Supplement: Supplementary file 1 — Supplementary Material 1 [file 41598_2025_87563_MOESM1_ESM.docx]

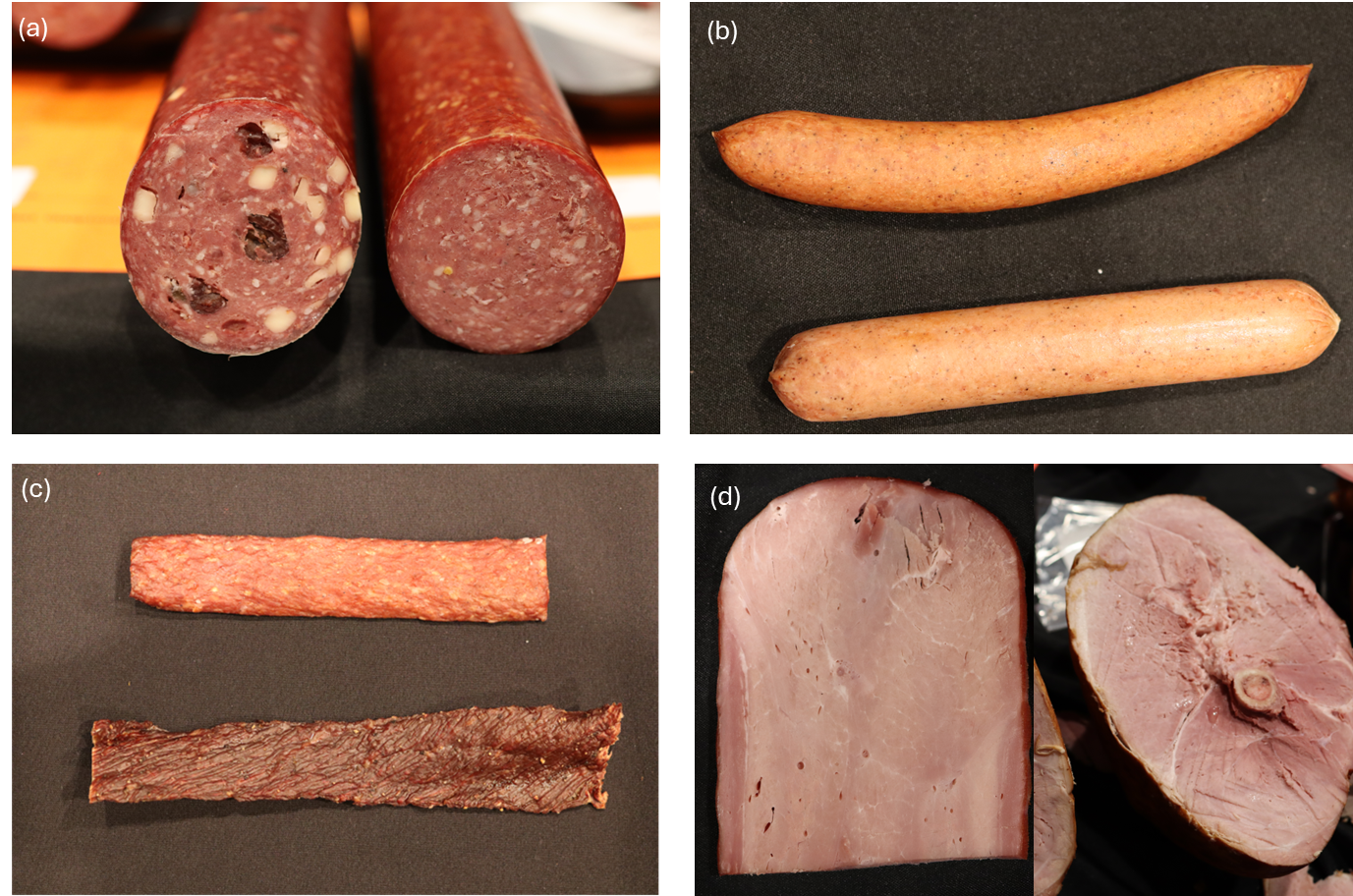


Figure S1. Example of processed meats (Photo taken during sample collection at Wisconsin Association of Meat Processor convention). (a) Specialty (left) vs. traditional (right) beef sticks. (b) Natural casing (top) vs. skinless (bottom) frankfurter. (c) Reconstructed (top) vs. whole muscle (bottom) beef jerky. (d) Boneless (left) vs. Bone-in (right) ham.


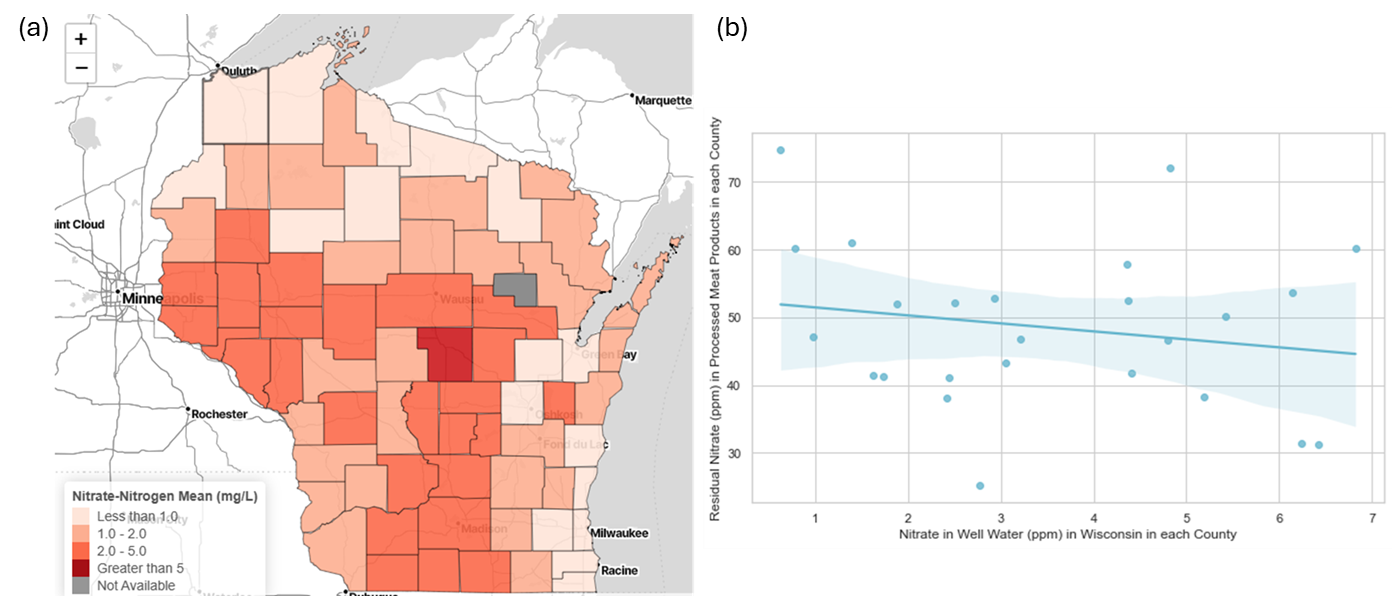


Figure S2. Correlation study on portable water and nitrate content in processed meats. (a) Heat map of nitrate content in each county of Wisconsin's potable water system. (b) The correlation (r < 0.25) between nitrate levels in potable water and processed meats produced in each county suggests a weak relationship.
